# Penile Cancer: The Importance to predict lymph node metastasis

**DOI:** 10.1590/S1677-5538.IBJU.2016.06.01

**Published:** 2016

**Affiliations:** Professor Associado da Unidade de Pesquisa Urogenital da Universidade do Estado de Rio de Janeiro Urologista do Hospital da Lagoa Federal, Rio de Janeiro; Hospital da Lagoa Federal, Hospital da Lagoa Federal, Rio de Janeiro, Brasil

The November-December 2016 issue of the International Braz J Urol presents original contributions with a lot of interesting papers in different fields: Bladder Cancer, BPH, Prostate Cancer, Renal stones, Renal Cell Carcinoma, Pediatric Urology, Peyronie Disease, Erectile Disfunction, Penile Cancer, Testicular torsion, Hypogonadism, and Hemorrhagic cystitis. The papers come from many different countries such as Brazil, USA, Turkey, India, China, Iran, UK, Netherlands, Germany, Pakistan and Chile, and as usual the editor's comment highlights some papers. We decided to comment the paper about a very usual topic in Brazil: Penile Cancer.

Doctor Aita and collegues from Brazil performed on page 1136 an interesting study about the prognostic factor in penile cancer. The authors evaluated some prognostic factors for global survival (GS) and cancer-specific survival (CSS) in a historical series of patients with penile cancer not submitted to lymphadenectomy and that did not show lymph node metastasis in a minimum follow-up of three years. The authors evaluated the clinical and pathologic characteristics of 163 patients with penile carcinoma and clinically negative inguinal lymph nodes followed for three or more years and their impact on global survival (GS) and cancer-specific survival (CSS) in the 10-year follow-up. Primary pathologic tumor stage (p=0.025) and the presence of high grade of tumor differentiation (p=0.018) were predictive of CSS. The presence of high grade tumor was an independent specific prognostic factor of death risk (RR 14.08; p=0.019). The authors concluded that a high histologic grade was an independent predictive factor of specific death risk in patients with penile carcinoma and clinically negative lymph nodes followed for three or more years.

Penile cancer is a rare neoplasia with low incidence in developed countries. In Brazil the incidence rate of penile cancer is 2.9 - 6.8/100,000 inhabitants, resulting in this country having one of the world's highest incidence rates for this neoplasia (1, 2). The most common sites of penile cancer metastasis are the superficial and deeper nodes of the inguinal and iliac region. Patients have inguinal groin masses in 58% of cases, and 40% have positive metastasis, even in small cancers such as T1C and T2 (3).

Penile lymphatic drainage parallels venous drainage, with a superficial system that drains the skin and a deeper system that drains the glans and corporal bodies. The superficial inguinal nodes are located just below the inguinal ligament and extend trough 4-5 cm of the saphenous hiatus. They are distributed in quarters set from the anastomosis between the saphena magna and femoral veins (4). The deeper inguinal nodes are located just below the fascia lata and medially to the saphena vein. Although small in number, these nodes are of extreme importance, since their venous drainage occurs through the superficial iliac veins (4). Extended Inguinal lymphadenectomy is the most useful and commonly performed surgery for staging and to cure inguinal metastasis in penile cancer cases. Although it is a widespread technique, post operatory complications often occur (3).

In the paper commented here we observed a interesting conclusion. The patients not submitted to inguinal lymphadenectomy and that did not regionally progress after three years, a small subgroup of patients died due to cancer. Main independent prognostic factor for CSS was the presence of high grade primary tumor. Patients not operated but with high grade tumors that refuse surgery comprise a high risk group and require a more diligent follow-up. The high histologic grade remains a risk factor for death due to penile carcinoma, even in sub-groups without lymph node metastasis. The great question in the treatment of penile cancer is: When we need to make the lymphadenectomy? This paper is very interesting and can help with this question in future researches.
